# Research hotspots and trends of spinal cord stimulation for neuropathic pain: a bibliometric analysis from 2004 to 2023

**DOI:** 10.1186/s13741-024-00433-4

**Published:** 2024-07-09

**Authors:** Liwen Zhang, Zhenhua Li, Haiyan Gu, Jinyan Chen, Yanping Zhang, Yuanyuan Yu, Hexiang Wang

**Affiliations:** 1 Discipline of Anesthesiology, Medical Department, Qingdao University, Qingdao, 266075 China; 2 Department of Anesthesiology, Qingdao Hiser Hospital Affiliated of Qingdao University (Qingdao Traditional Chinese Medicine Hospital), Qingdao, 266034 China; 3 Department of Pathology, The Affiliated Hospital of Qingdao University, Qingdao, 266555 China; 4 Department of Nephrology, Qingdao Hiser Hospital Affiliated of Qingdao University (Qingdao Traditional Chinese Medicine Hospital), Qingdao, 266034 China; 5 Department of Pathology, Qingdao Hiser Hospital Affiliated of Qingdao University (Qingdao Traditional Chinese Medicine Hospital), Qingdao, 266034 China

**Keywords:** Spinal cord stimulation, Neuropathic pain, Treatment, Bibliometric, Visualization

## Abstract

The purpose of this study is to systematically analyze the development trend, research hotspots, and future development direction on the treatment of neuropathic pain (NP) with spinal cord stimulation through bibliometric method. We extracted the literature related to the treatment of NP with spinal cord stimulation from January 2004 to December 2023 from the Web of Science database. As a result, a total of 264 articles were retrieved. By analyzing the annual published articles, authors, countries, institutions, journals, co-cited literature, and keywords, we found that the count of publication in this field has been experiencing an overall growth, and the publications within the past 5 years accounted for 42% of the total output. Experts from the United States and the UK have made significant contributions in this field and established a stable collaborative team, initially establishing an international cooperation network. *Pain* is the frequently cited journal in this field. The study on spinal cord stimulation therapy for NP especially the study on spinal cord stimulation therapy for back surgery failure syndrome (FBSS) and its potential mechanisms are the research hotspots in this field, while the study on novel paradigms such as high-frequency spinal cord stimulation and spinal cord burst stimulation represents the future development directions. In short, spinal cord stimulation has been an effective treatment method for NP. The novel paradigms of spinal cord stimulation are the key point of future research in this field.

## Introduction

Neuropathic pain (NP) refers to pain caused by lesions or diseases of the somatosensory nervous system and characterized by spontaneous pain and paresthesia in the pain area. In severe cases, it can lead to mental symptoms such as depression and anxiety (Bates et al. 2019; Mitsikostas et al. 2022; Yan et al. 2017). According to the International Association for the Study of Pain (IASP), the prevalence of NP is between 6.9 and 10%, and the global population of NP is about 500 to 700 million, accounting for 1/5 to 1/4 of patients with chronic pain (Bouhassira 2019). However, the complex pathogenesis of NP makes us lack of effective treatment methods. Drug therapy has always been the main means to relieve NP, but opioid drugs could cause addiction, tolerance, and dependence, while commonly used anticonvulsants, antidepressants, and other drugs could cause dizziness, drowsy, severe headache, hypertension, and other adverse reactions, all of which could not meet the needs of clinical treatment. Therefore, alternative therapies have been developed. Spinal cord stimulation may offer a rescue option when conventional treatments produce unacceptable adverse effects or do not provide adequate pain relief. It can be used alone or in combination with other modalities. The continuous development of spinal cord stimulation technology makes it more and more popular, and it is expected to become an effective means of treating NP in the future.

Bibliometric which appeared in the early twentieth century is a subject that uses mathematical and statistical techniques to quantify and analyze literatures. We can obtain detailed information such as authors, countries, journals, institutions, keywords, and references by the bibliometric and use graphics and visual results to supplement literatures analysis with the help of modern computer technology.

At present, there is little study of the analysis of development trend in the treatment of NP with spinal cord stimulation. The aim of this study is to analyze the literatures published from 2004 to 2023 which related to the treatment of NP with spinal cord stimulation by bibliometric and then systematically understand the research trends, hotspots, and future development directions in this field.

## Literature and methods

### Literature sources and search methods

In order to ensure the retrieval data comprehensively and accurately, we selected the Web of Science (core collection) as the data source and SCI-EXPANDED and SSCI as the index. We used the advanced retrieval; the search formula was set as TS = "neuropathic pain" AND TS = ("Spinal cord stimulation" OR SCS) AND TS = (cure OR treat OR remedy OR therapy OR treatment OR "therapeutic schedule" OR "therapeutic regimen" OR "therapeutic method" OR therapies); the time span was from January 1, 2004, to December 31, 2023; the type of language was set in English; and the type of document was selected as article and review.

It needs to be mentioned that the aforementioned formula was as inclusion criteria utilized for preliminary screening, and the secondary screening was conducted based on exclusion criteria which requiring the keywords such as spinal cord stimulation and neuropathic pain be presented in the abstract, while the content of the articles was not pertain to the application of spinal cord stimulation in treating neuropathic pain. Finally, all records of the literature such as title, author, abstract, and citation were exported in TXT format excluding duplicate literature.

### Research methods

**Fig. 1:**
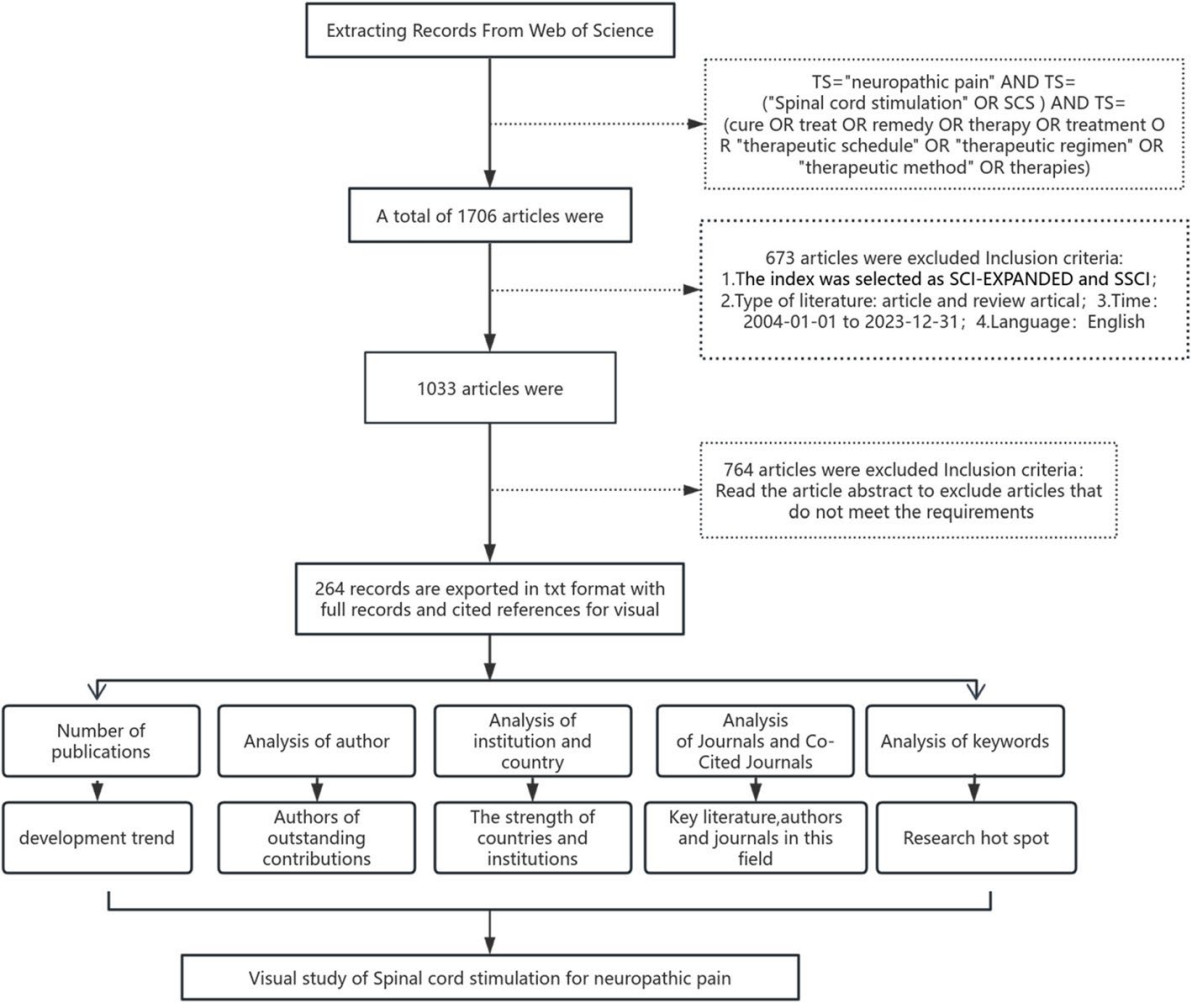
Flowchart of article screening and analysis

CiteSpace is a scientific literature analysis tool jointly developed by Dr. Chaomei Chen, a Chinese-American scholar, and WISE Laboratory. Based on co-citation analysis and pathfinding network algorithm, the software visualizes data samples, presents the evolution process of the field at specific values, and visualizes the relationship between documents in the way of scientific knowledge map. It can not only help users clarify the past research trajectory, research status, and hot topics in a certain field but also reveal the future development direction of the field. VOSviewer is a metrological analysis software developed by Nees Janvan Eck and Ludo Waltman of Leiden University in the Netherlands to construct and visualize network maps. It has the characteristics of strong visualization ability and is suitable for large-scale sample data. The software supports users to create knowledge graphs through VOS mapping technology and VOS clustering technology. At the same time, it provides four kinds of graph browsing methods, such as label view, density view, cluster view, and scatter view, as well as zooming and scrolling functions to help users draw and observe knowledge graphs. In our study, CiteSpace and VOSviewer were used for visual analysis of authors, countries, institutions, journals, keywords, and co-cited literature. Figure 1 illustrates the specific analysis process of this study.

## Results

### Analysis of the number of annual publications

**Fig. 2:**
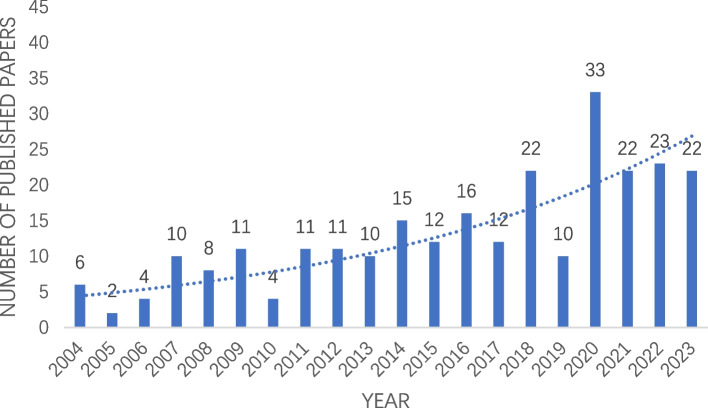
Annual publication number related to spinal cord stimulation in the treatment of NP

As shown in Fig. 2, a total of 264 articles were retrieved in this study which comprising 164 observational studies, 55 randomized controlled trials, 37 narrative reviews, and 8 systematic reviews. From 2004 to 2023, the number of publications related to spinal cord stimulation in the treatment of NP showed an overall upward trend, and the year 2020 with the largest number of publications (33 articles), followed by 2022 (23 articles). In the past 5 years, there were 110 articles related to spinal cord stimulation in the treatment of NP published, accounting to 42% of the total number of publications.

### Analysis of authors and co-cited authors

**Fig. 3:**
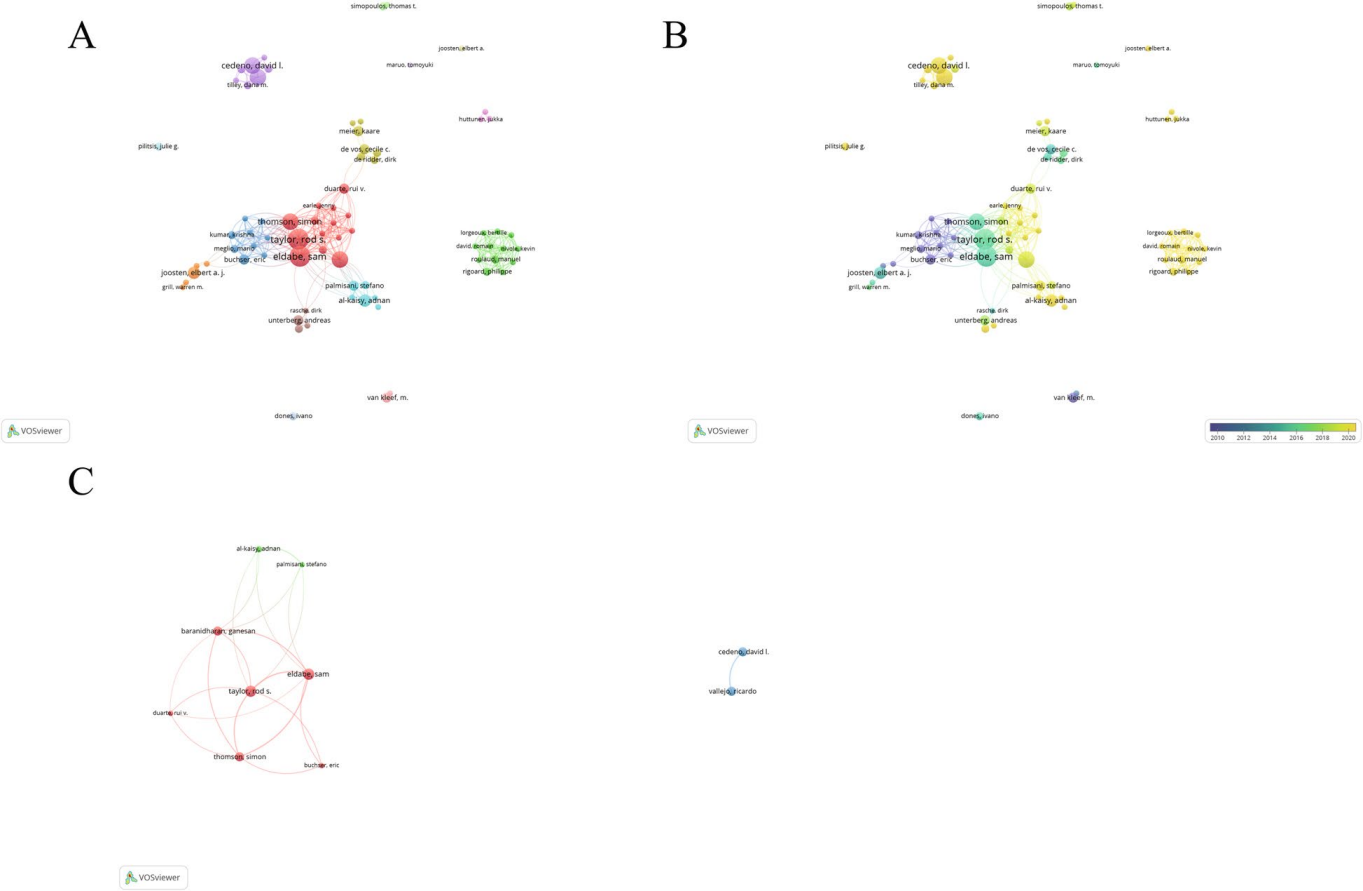
Author co-occurrence analysis. **A** Clustering view of author co-occurrence networks. **B** Author collaboration network label view. **C** Top author collaboration networks

A total of 1180 authors contributed 264 articles. In terms of the number of publications, top 10 authors were listed in Table 1. Eldabe, Sam and Taylor, Rod S. each published 10 articles; the former was cited 1523 times and the latter 1442 times (Taylor et al. 2005; Cameron 2004; Turner et al. 2004). Buchser and Eric had the highest number of citations per article (289.6 times). Six out of the top 10 authors were from the UK, followed by 2 authors from the United States. Furthermore, we selected 75 authors with more than 3 articles for visual analysis (Fig. 3) and found a stable cooperative group had been formed in this field. The collaborative relationships among top authors in the field were shown in Fig. 3C.

**Table 1:** Top 10 authors with published articles on spinal cord stimulation therapy for NP

Rank	Author	Counts	Citations	Country
1	Eldabe, Sam	10	1523	UK
2	Taylor, Rod S	10	1442	UK
3	Thomson, Simon	8	1435	UK
4	Baranidharan, Ganesan	8	205	UK
5	Cedeno, David L	8	140	USA
6	Vallejo, Ricardo	8	140	USA
7	Al-Kaisy, Adnan	6	145	UK
8	Buchser, Eric	5	1448	Switzerland
9	Palmisani, Stefano	5	139	Italy
10	Duarte, Rui V	5	85	UK

**Fig. 4:**
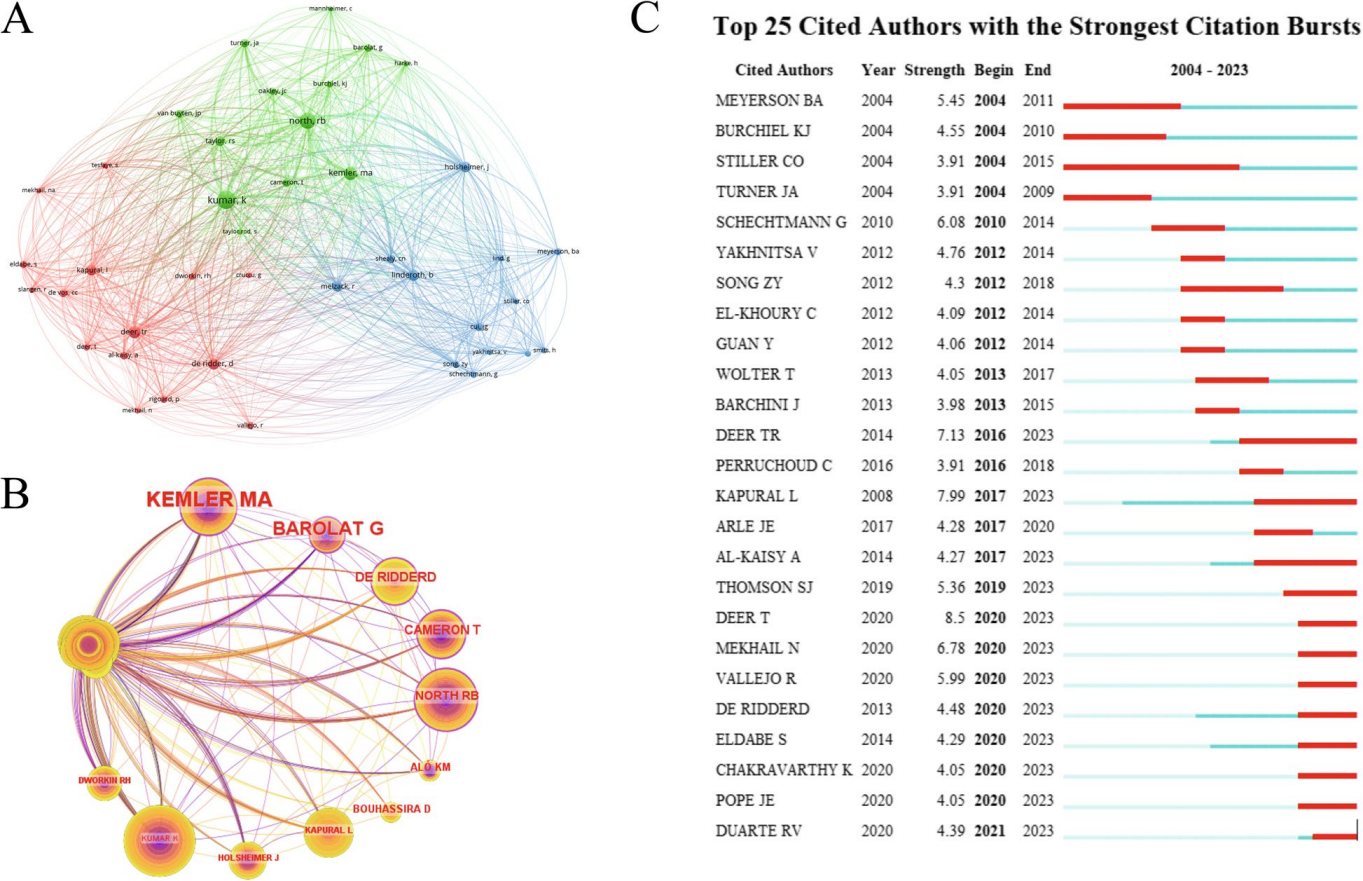
Co-occurrence analysis of co-cited author. **A** Cluster view of co-cited authors’ collaborative network. **B** Co-occurrence network of co-cited author centrality. **C** Top 25 cited authors with the strongest citation bursts

A total of 4774 co-cited authors were included. We took visual analysis on the authors whose articles be cited ≥ 20 times. The results were presented in Fig. 4, and Table 2 displayed the top 10 authors for co-citation and centrality. Among them, Kumar, K. emerged as the most frequently cited author (238 citations) (Kumar et al. 2006, 2008, 2007a), while Kemler, M. A. exhibited the highest centrality value (0.19). These two authors hold significant influence within the field. In terms of citations, the three authors with the highest outbreak intensity were Deer, T. (2020–2023), Kapural, L. (2017–2023), and Deer, T. R. (2016–2023). It is highly probable that these three authors will continue their research endeavors in this field.

**Table 2:** Top 10 co-cited authors for the treatment of NP with spinal cord stimulation

Rank	Co-cited author	Fre	Co-cited author	Centrality
1	Kumar, K	238	Kemler, M. A	0.19
2	North, R. B	184	Barolat, G	0.16
3	Kemler, M. A	127	De, Ridder D	0.12
4	Deer, T. R	100	Cameron, T	0.11
5	Linderoth, B	88	North, R. B	0.10
6	Kapural, L	78	Bouhassira, D	0.09
7	Melzack, R	75	Alo, K. M	0.09
8	Holsheimer, J	75	Kapural, L	0.08
9	Taylor, R. S	70	Holsheimer, J	0.08
10	Barolat, G	46	Kumar, K	0.07

### Analysis of countries and institutions

**Fig. 5:**
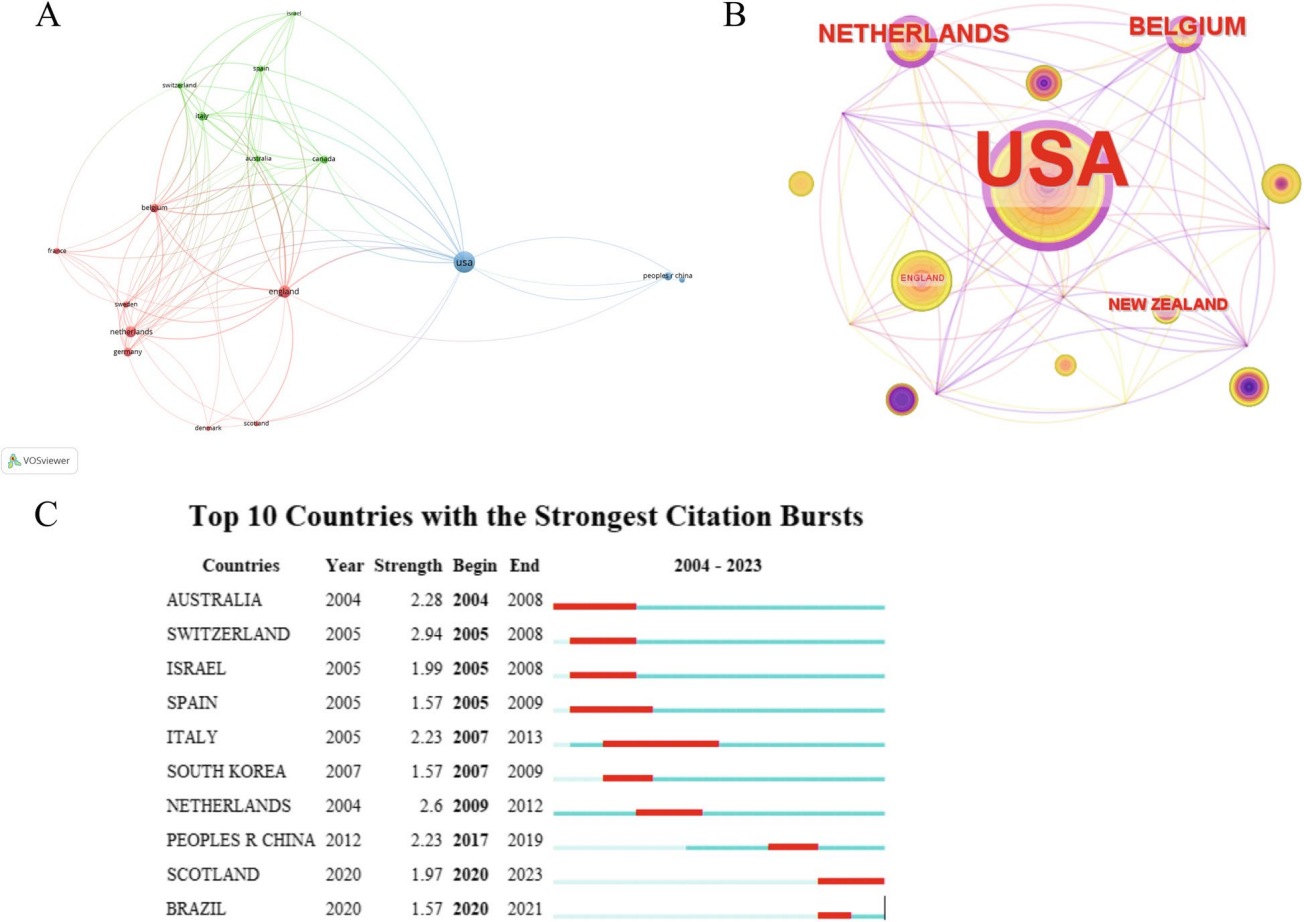
Country co-occurrence analysis. **A** Cluster view of national cooperative networks. **B** Country-centric co-occurrence networks. **C** Top 10 countries with the strongest citation bursts

There were 264 articles to be published by research teams from 35 countries. As shown in Fig. 5, we conducted a visual analysis on 18 countries with ≥ 5 publications. Table 3 presented the top 10 countries in terms of publication volume and centrality. Among them, the United States emerged as the leading country with the highest number of publications (113 articles) and centrality (0.36), exhibiting a significant gap compared to the second-ranked country, thereby reflecting its robust scientific research prowess and influence in this domain. Switzerland exhibited the highest outbreak intensity during the period from 2005 to 2008, while Scotland demonstrated notable outbreak intensity in recent years (2020–2023), potentially positioning it as a pivotal country for future advancements in this field.

**Table 3:** Top 10 countries studying the treatment of NP with spinal cord stimulation

Rank	Country	Counts	Country	Centrality
1	USA	113	USA	0.36
2	UK	37	Netherlands	0.12
3	Netherlands	29	Belgium	0.12
4	Italy	18	New Zealand	0.08
5	Germany	18	UK	0.05
6	Belgium	18	Canada	0.02
7	China	17	France	0.02
8	Canada	17	Switzerland	0.01
9	France	11	Australia	0.01
10	Australia	11	Germany	0.01

**Fig. 6:**
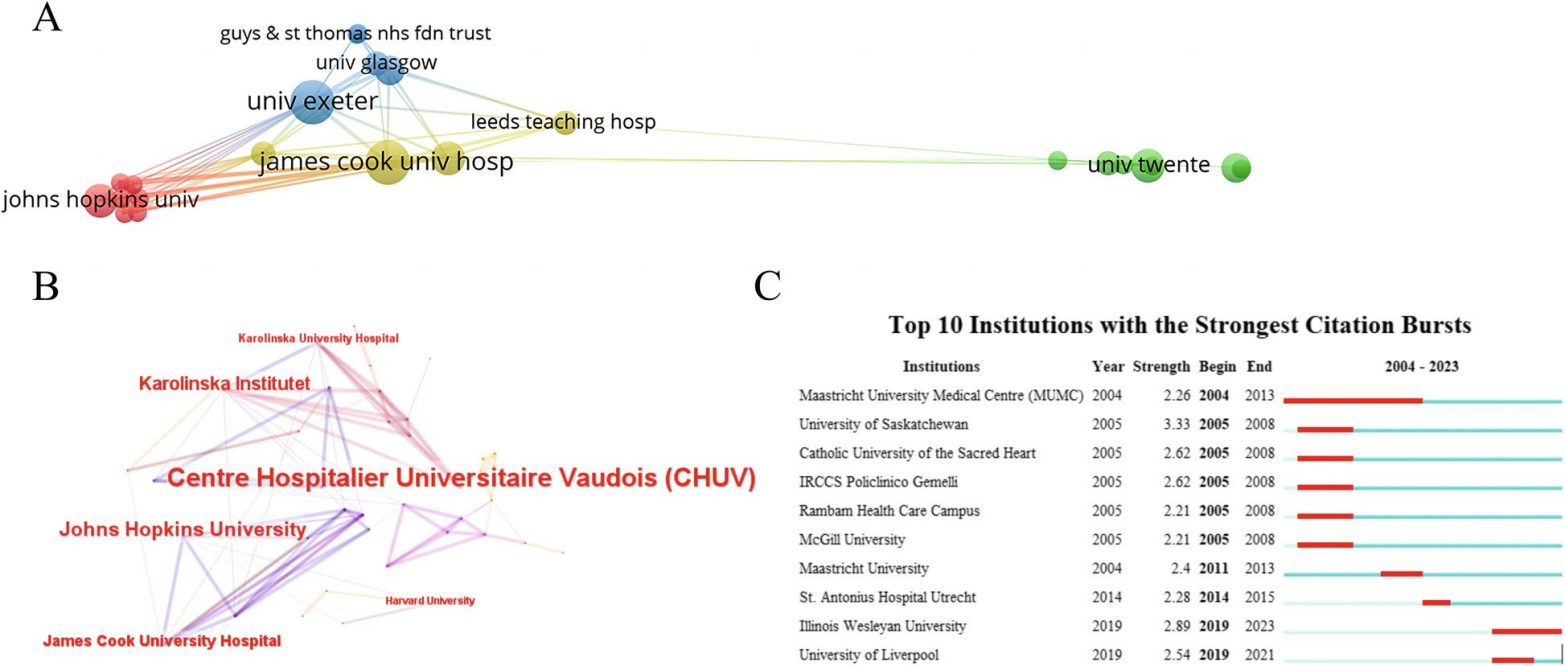
Institutional co-occurrence analysis. **A** Cluster view of institutional cooperation networks. **B** Institutional centrality co-occurrence network. And **C** top 10 institutions with the strongest citation bursts

A total of 515 institutions participated in this field. We conducted a visual analysis of institutions with ≥ 4 publications, and the results were presented in Fig. 6. Table 4 displayed the top 10 institutions in terms of publication volume and centrality. Maastricht University from the Netherlands emerged as the research institution with the highest publication volume, while among the top 10 research institutions, both the Netherlands and the United States had 3 respectively. The most core institution was the University of Vaduat Medical Center in Switzerland, and the United States had a leading position in this research field accompanied by 3 out of the top 10 institutions being from the United States. In addition, the University of Saskatchewan exhibited the highest outbreak intensity within this field (2005–2008), whereas the University of Illinois Wesleyan demonstrated peak outbreak intensity in recent years (2019–2023).

**Table 4:** Top 10 research institutions for the treatment of NP with spinal cord stimulation

Rank	Institutions	Counts	Country	Institutions	Centrality	Country
1	Maastricht University	13	Netherlands	Centre Hospitalier Universitaire Vaudois (CHUV)	0.09	Switzerland
2	Johns Hopkins University	12	USA	Johns Hopkins University	0.07	USA
3	James Cook University Hospital	10	Australia	Karolinska Institutet	0.06	Sweden
4	Harvard University	8	USA	James Cook University Hospital	0.05	Australia
5	Maastricht University Medical Centre (MUMC)	7	Netherlands	Karolinska University Hospital	0.04	Sweden
6	University of Twente	7	Netherlands	Cleveland Clinic Foundation	0.03	USA
7	Illinois Wesleyan University	7	USA	CHU de Nantes	0.03	France
8	Karolinska Institutet	6	Sweden	Case Western Reserve University	0.03	USA
9	Catholic University of the Sacred Heart	6	Italy	Catholic University of the Sacred Heart	0.03	Italy
10	University of Exeter	6	UK	IRCCS Policlinico Gemelli	0.03	Italy

### Analysis of journals

**Fig. 7:**
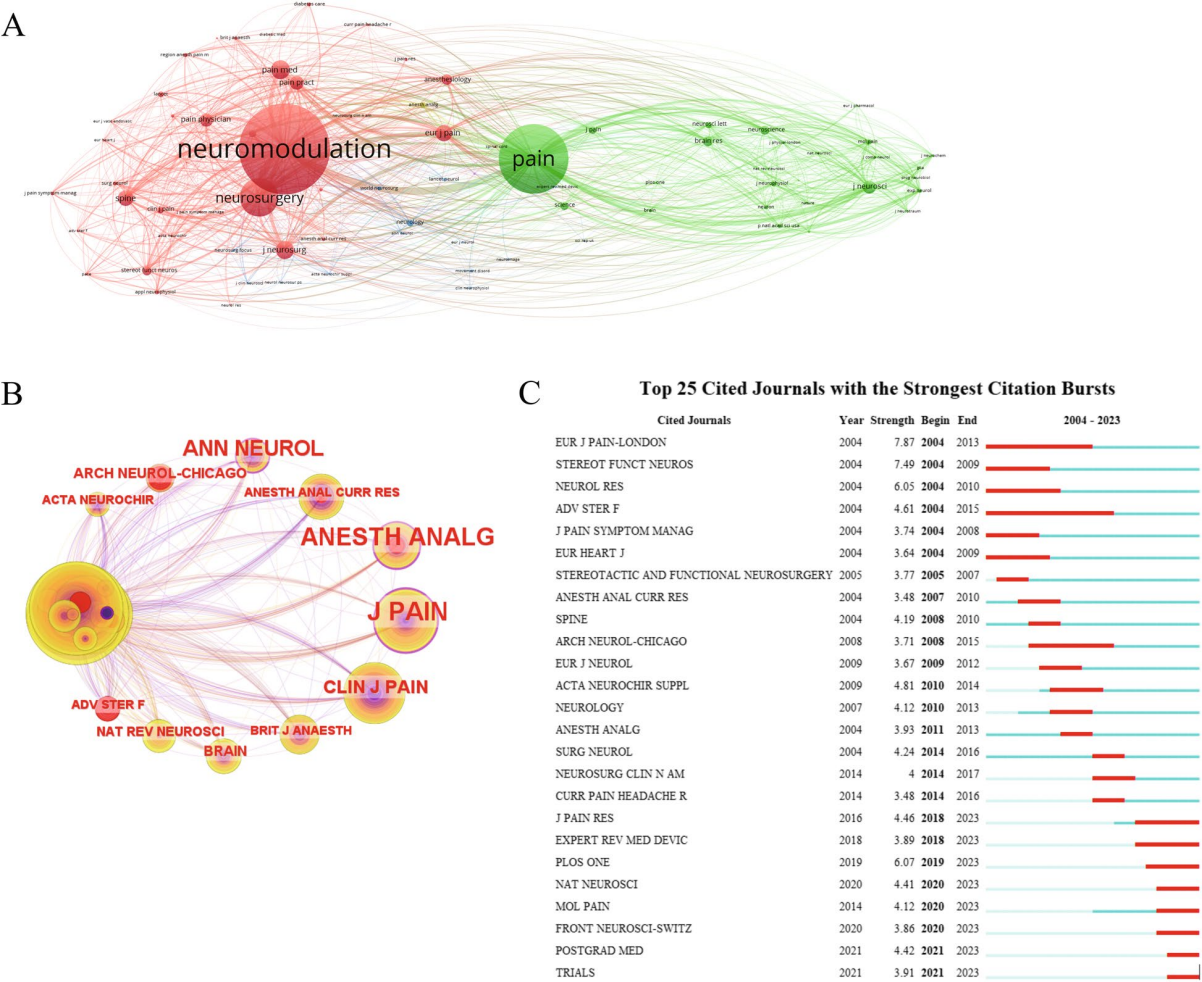
Co-occurrence analysis of co-cited journals. **A** Cluster view of co-cited journals. **B** Co-occurrence network of co-cited journal centrality. **C** Top 25 cited journals with the strongest citation bursts.

The top 10 journals for co-citation and centrality were presented in Table 5. The journal of *Pain* emerged as the most frequently cited publication, garnering a total of 197 citations, and it is closely followed by the journal of *Neuromodulation* with 190 citations. The *Journal of Pain* and *Anesthesia and Analgesia* exhibited the highest centrality scores (both at 0.14). In addition, *the European Journal of Pain-London* (2004–2013) stood out as the greatest intensity of co-cited outbreaks journal, while *PLOS One* currently held the highest intensity of outbreaks (2019–2023) (Fig. 7). All of the above reveals that pain, anesthesia, and neurology are hotspots and future research directions in the field of treating NP.

**Table 5:** Top 10 cited journals for the treatment of NP with spinal cord stimulation

Rank	Co-cited journal	Counts	IF (2022)	Co-cited journal	Centrality	IF (2022)
1	*Pain*	197	7.4	*J Pain*	0.14	4
2	*Neuromodulation*	190	2.8	*Anesth Analg*	0.14	5.9
3	*Neurosurgery*	168	4.8	*Ann Neurol*	0.12	11.2
4	*Pain Med*	119	3.1	*Clin J Pain*	0.1	2.9
5	*J Neurosurg*	113	4.1	*Brain*	0.08	14.5
6	*Pain Pract*	106	2.6	*Arch Neurol-Chicago*	0.08	-
7	*Eur J Pain*	99	3.6	*J Neurosci*	0.07	5.3
8	*Spine*	86	3	*Adv Ster F*	0.07	-
9	*Anesthesiology*	84	8.8	*Nat Rev Neurosci*	0.07	34.7
10	*Pain Physician*	81	3.7	*Acta Neurochir*	0.07	2.4

### Analysis of co-cited literature

**Fig. 8:**
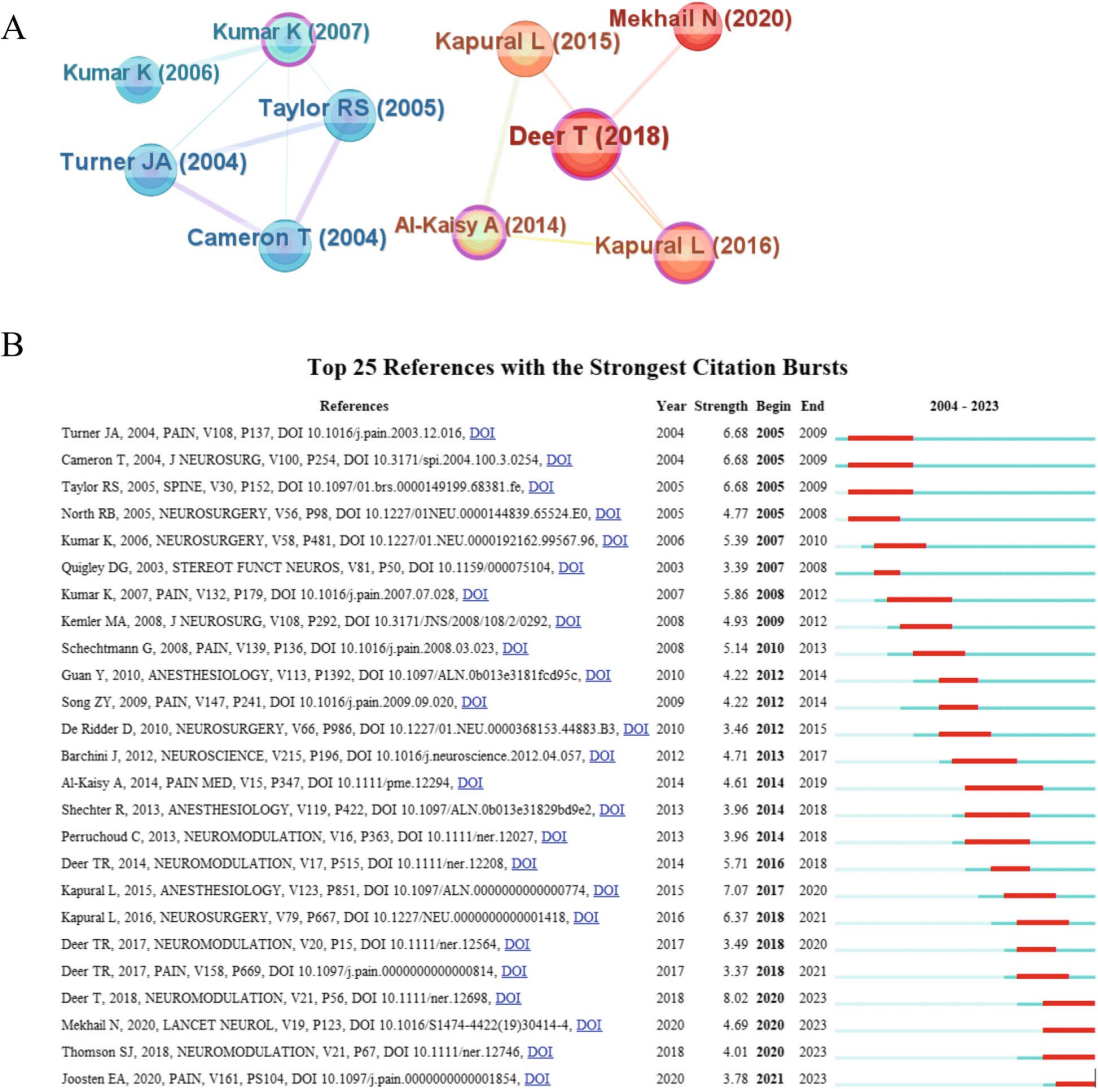
Co-occurrence analysis of cited articles. **A** Co-occurrence network of top 10 cited articles. **B** Top 25 references with the strongest citation bursts

In our study, the top 10 articles with co-citation frequency were shown in Table 6, among which the articles from Deer, T. had the highest citation frequency (22 times) and citation outbreak intensity (8.02, 2020–2023). Both Kapural, L. and Kumar, K. had 2 in the top 10 articles respectively. Among these 10 articles, there were totally 5 randomized controlled trials, 2 observational studies, 2 systematic analysis, and 1 literature review. Therein, 4 out of 5 randomized controlled trials focused on the new paradigm of spinal cord stimulation which encompasses burst spinal cord stimulation, high-frequency spinal cord stimulation, and closed-loop spinal cord stimulation, while the remaining one compared the therapy effects of spinal cord stimulation with drug for NP. Furthermore, the observational studies, systematic analysis, and literature reviews were all relevant to investigate the treatment for NP with spinal cord stimulation especially on failed back surgery syndrome (FBSS). All of the above indicate that there is an increasing attention towards the new paradigm of spinal cord stimulation as well as its application in treating chronic pain especially on FBSS. Figure 8A presented the co-citation analysis of the top 10 cited articles (node size represents citation frequency; node color represents proximity to current time with warmer colors indicating closer proximity). We can see that 5 out of 10 articles were published before 2014 which primarily focused on evaluating the efficacy of spinal cord stimulation in treating chronic pain and comparing it with drug therapy, while the other 5 articles were published after 2014 which mainly centered on studying the novel paradigms in spinal cord stimulation which reflect technology advancements within this field. Figure 8B listed the top 25 articles with the highest citation outbreak intensity.

**Table 6:** Top 10 articles with high citation rates

Rank	Author	Counts	Centrality	Bursts
1	Deer, T. (2018)	22	0.12	8.02
2	Kapural, L. (2016)	15	0.18	6.37
3	Kapural, L. (2015)	15	0.02	7.07
4	Cameron, T. (2004)	14	0.06	6.68
5	Turner, J. A. (2004)	14	0.02	6.68
6	Taylor, R. S. (2005)	14	0.02	6.68
7	Mekhail, N. (2020)	13	0.05	4.69
8	Kumar, K. (2007)	12	0.29	5.86
9	Al-Kaisy, A. (2014)	11	0.43	4.61
10	Kumar, K. (2006)	11	0.04	5.39

### Analysis of keywords

**Fig. 9:**
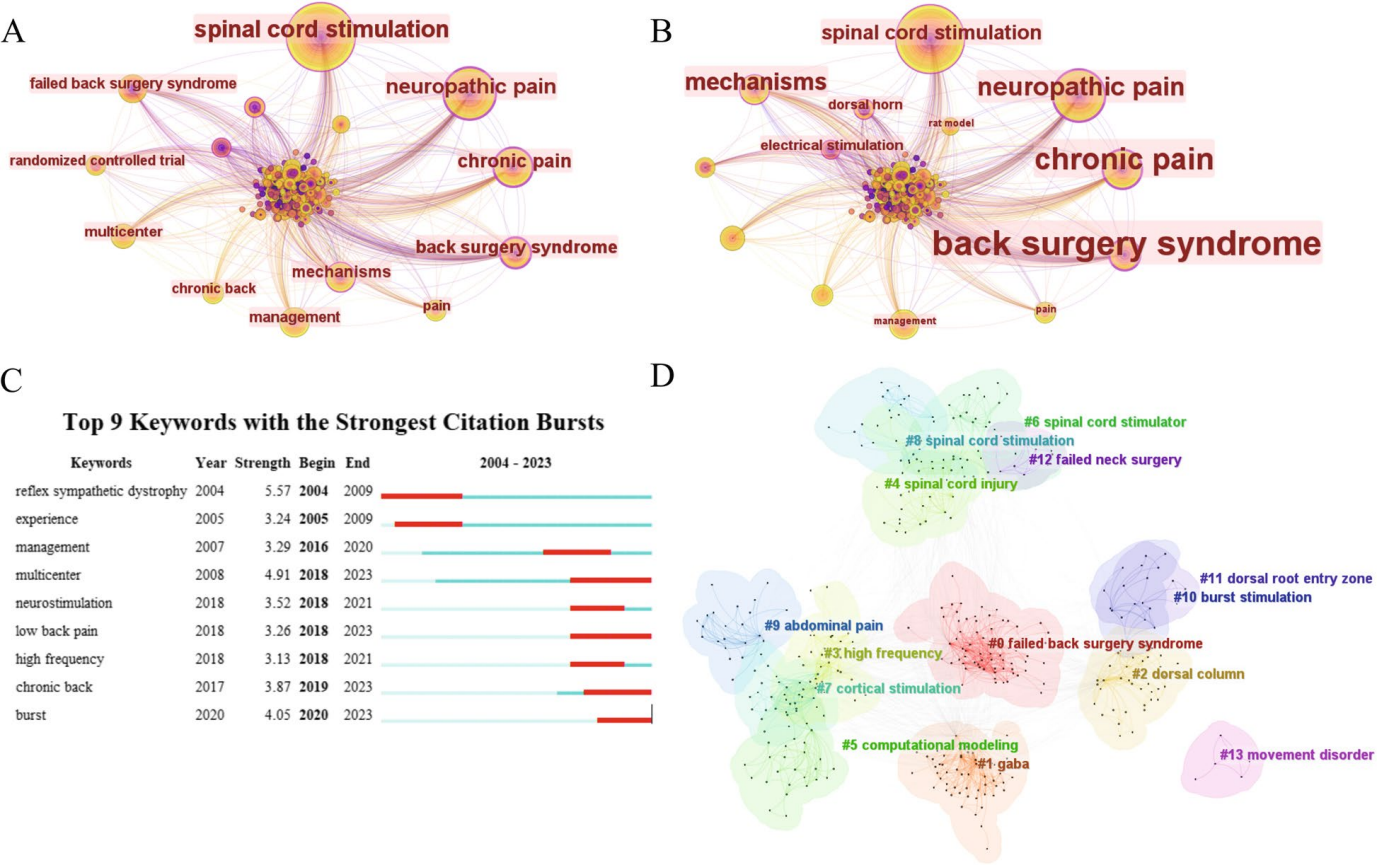
Keyword co-occurrence analysis. **A** Keyword co-occurrence network. **B** Keyword centrality co-occurrence network. **C** Top 9 keywords with the strongest citation bursts. **D** Keyword clustering network

As shown in Fig. 9A, a total of 634 keywords were retrieved, and Table 7 showed the top 10 keywords with the highest number of occurrences and centrality. Besides spinal cord stimulation and neuropathic pain, the keywords such as chronic pain, back surgery syndrome, and mechanisms were the three most frequently used keywords which were also the three most central keywords. All of the above indicated that chronic pain, back surgery syndrome, and mechanisms were the research hotspots in this field, and most of the research was related to them. Figure 9C showed the 9 keywords with the higher burst intensity, of which reflex sympathetic dystrophy (5.57) was the keyword with the highest burst intensity. In recent years, the keywords with the highest burst intensity were multicenter (4.91), low back pain (3.26), chronic back (3.87), and burst (4.05). We clustered the retrieved keywords according to their connection tightness and obtained a total of 13 cluster labels (Fig. 9D). The smaller the number, the more keywords were included in the cluster. The most common keyword was failed back surgery syndrome (#0), followed by gaba (#1) and dorsal column (#2).

**Table 7:** Top 10 keywords in the treatment of NP with spinal cord stimulation

Rank	Keywords	Counts	Keywords	Centrality
1	Spinal cord stimulation	173	Back surgery syndrome	0.28
2	Neuropathic pain	111	Chronic pain	0.25
3	Chronic pain	56	Neuropathic pain	0.21
4	Back surgery syndrome	48	Mechanisms	0.19
5	Mechanisms	38	Spinal cord stimulation	0.16
6	Management	25	Electrical stimulation	0.12
7	Failed back surgery syndrome	24	Dorsal horn	0.11
8	Multicenter	21	Management	0.09
9	Pain	18	Rat model	0.09
10	Randomized controlled trial	17	Pain	0.09

## Discussion

In this study, we conducted a visual analysis of articles on the treatment of NP with spinal cord stimulation and found that in the past two decades, the number of publications in this field continues to increase, and more and more researchers are paying attention to the development of spinal cord stimulation for the treatment of NP. *Pain*, *Neuromodulation*, and *Neurosurgery* emerged as the three most frequently cited journals in this domain which suggest that spinal cord stimulation for NP is being extensively studied within these domains. Eldabe, Sam and Taylor, Rod S. are the authors with the highest number of publications; it can be seen from the author collaboration network that they have a close collaborative relationship, and the article which has the most citations comes from their collaborative study. For instance, in 2007, they conducted a multicenter randomized controlled trial on patients who suffer from failed back surgery syndrome (FBSS), and the results showed that spinal cord stimulation can better alleviate the pain of patient and improve health-related quality of life and functional abilities compared to medication alone after a 1-year follow-up (Hong et al. 2020; Kumar et al. 2005). Subsequently, in 2008, they published another collaborative study focusing on the sustained treatment effects of spinal cord stimulation wherein patients reported enduring pain relief along with significant improvements in functional ability even after 24 months of treatment duration. Additionally, at the same year, one article they published suggests that despite increased resource consumption costs associated with spinal cord stimulation technology, there is a noteworthy enhancement in patients’ overall quality of life (Manca et al. 2008). Interestingly, His article has been cited 355.25 times, ranking first with a total of 238 citations. In 2005, Kumar, K. conducted a multicenter study on patients with FBSS. Subsequently, in 2007, due to the lack of formal consensus on optimal strategies for reducing spinal cord stimulation (SCS) complications, an international panel of experts led by Kumar, K comprehensively reviewed the existing literatures on SCS complications and drafted practical recommendations which aimed at minimizing these complications risks. The publication of “Avoiding complications from spinal cord stimulation: practical recommendations from an International panel of experts” offers valuable guidelines for physicians to enhance their SCS techniques, thereby improving treatment outcomes (Kumar et al. 2007b; Hofmeister et al. 2020). These recommendations proved particularly beneficial for patients undergoing SCS treatment while also laying the groundwork for further advancements in spinal cord stimulation.

Spinal cord stimulation has been reported to effectively alleviate various types of chronic NP which including FBSS, complex regional pain syndrome, and chronic peripheral neuropathy. Among the 264 articles we retrieved, 54 articles were related to the treatment of FBSS with spinal cord stimulation, while 45 articles were related to complex regional pain syndrome, all of which indicating the widespread application of spinal cord stimulation for treating chronic pain especially in FBSS. However, the mechanism underlying the pain relief through spinal cord stimulation therapy is intricate (Joosten and Franken 2020). During the past two decades, mechanisms have been a prominent focus, with high-frequency being the primary keyword and following closely by GABA and dorsal column as the second and third largest clusters of keywords, all of which indicates that research on the mechanism of spinal cord stimulation has been a significant area of interest in this field. The most widely accepted theory is known as the gating theory, which proposes that non-painful input closes the “neural gate” for pain signals, thereby preventing their perception by the brain (Deer et al. 2017; Levy et al. 2020; Deer et al. 2018; Mekhail et al. 2020a; Graham et al. 2019). Traditional tonic SCS directly stems from this gating theory concept. Joosten E. A. et al. elucidated extensively upon both the mechanism of action and limitations of tonic SCS while also providing prospects for future applications within this new paradigm of SCS therapy.

High-frequency spinal cord stimulation and burst spinal cord stimulation represent the new paradigm in this field. In recent years, in a keyword burst analysis, it is evidently that high frequency and burst have garnered significant attention from researchers as reflected by their high burst intensity. High-frequency spinal cord stimulation (HFSCS) is applied at frequencies above 1 kHz, up to 10 kHz, with a pulse width of approximately 30 μs and an amplitude typically ranging from 1 to 5 mA. In comparison to traditional tetanic spinal cord stimulation, HFSCS effectively alleviates pain without inducing any foreign sensations, thereby significantly addressing the needs of patients (Kapural et al. 2016, 2015; Al-Kaisy et al. 2014; Chakravarthy et al. 2018). Burst spinal cord stimulation was proposed in 2010 and is currently the most prominent keyword in recent years with regard to burst intensity. The burst waveform consists of five closely packed single-phase spikes, delivered at a burst pattern of 40 Hz and a burst frequency of 500 Hz, with a pulse width of 1 ms, spike interval of 1 ms, and constant current mode (Ridder et al. 2010). In 2018, Deer, T. et al. demonstrated the safety and effectiveness of burst stimulation by using a device that can provide both traditional tetanic stimulation and burst stimulation to patients (Deer et al. 2018). They found that burst stimulation was superior to traditional tetanic stimulation in the treatment of chronic pain. This literature currently holds the highest citation count within this field.

In addition to new paradigms and stimulation sites, the investigation of novel devices for SCS also constitutes a focal point in research. These new devices have been extensively discussed and referenced in recent literature. Deer, T. et al. employed a multimode stimulation device in their study and demonstrated its significant advantages. Similarly, Mekhail, N. et al. elucidated closed-loop stimulation which is controlled by an innovative spinal cord stimulation system offered patients superior and more clinically meaningful pain relief over a period of up to 12 months compared to open-loop spinal cord stimulation (Mekhail et al. 2020b).

It is speculated that new paradigms, alternative stimulation sites, and advanced devices of SCS will be the research directions in this field. The continuous improvement of SCS technology will gradually overcome its limitations and become an indispensable approach for treating NP.

## Conclusion

In this study, we used bibliometrics to comprehensively analyze the research on spinal cord stimulation in the treatment of NP which include the depth analysis of publications, authors, institutions, countries, journals, references, and keywords. The results indicate that the therapy of spinal cord stimulation for NP has received increasing attention from scholars, and stable cooperative groups have been formed among scholars from various countries. The United States is the country with the greatest influence in this research field. Pain is the journal with the most citations. The treatment of chronic pain in the lower back and the study of the analgesic mechanism of spinal cord stimulation are current research hotspots. Dorsal root ganglion, high-frequency spinal cord stimulation, and burst spinal cord stimulation technology will be the future development direction, and its treatment of chronic low back pain especially FBSS will be the main research hotspots in the future.

## Data Availability

No datasets were generated or analysed during the current study.

## Abbreviations

NP: Neuropathic pain
IASP: International Association for the Study of Pain
FBSS: Failed back surgery syndrome
SCS: Spinal cord stimulation
HFSCS: High-frequency spinal cord stimulation
